# Discussion of Dr. Tignor’s Paper

**Published:** 1907-05

**Authors:** 


### DISCUSSION OF DR. TIGNOR'S PAPER.

---

DR. C. M. BARNWELL said that the dental profession has awakened to the fact that to render the greatest good to the public they must see their patients at an earlier date. The necessity of treating the permanent teeth, alveolar abscesses, Rigg's disease, etc., is generally recognized. With children, these matters assume a greater importance than in adults, especially as regards the six-year molars, which are frequently injured before parents realize the danger, and much disfiguing results. Taken early. they may be filled and preserved. This is of great importance, for the temporary teeth establish the position of the permanent ones, and, if they are lost, result in an unsightly line of the teeth.

Dentists need the co-operation of physicians in order to get the patients early, before the damage is done. The parents should be impressed with the fact that it is not the candy and sweets that cause decay of the teeth, but that the part left in the mouth to decompose and form acids, is the cause. They should be urged to keep the mouths and teeth clean rather than to deprive the children of these things.

DR. KIME also thought there should be a closer relation between the physician and the dentist, and that parents should be instructed in the care of their children's teeth and mouths, as well as diet, etc.

DR. ARMSTRONG referred to the fact that he had a search made at the Home of the Friendless to find how many tooth-brushes were used in that institution. Only one was found on the place, and it belonged to a colored woman. Three things are chiefly responsble for indigestion: 1st, lack of suitable molar teeth for thorough mastication of food; 2nd, decayed teeth; 3rd, Riggs' disease, which is very frequent. All of these should receive appropriate treatment in the management of chronic indigestion.

DR. MCRAE mentioned the importance of this subject to the surgeon, as decayed teeth and a dirty, infected mouth are very frequently the cause of ether pneumonia. He makes it a rule to have the teeth attended to and the mouth thoroughly cleansed wherever practicable before giving an anesthetic.

DR. TOEPEL called attention to the marked improvement in the care of the children's teeth since last year, when they were first instructed as to the importance of this matter.

DR. TIGNOR (closing) thanked the gentlemen present for the kind reception of his paper, which was only a suggestive one. Good results in his privatep ractice, however, had followed the application of the principles outlined.

